# Ligand-human serum albumin analysis: the near-UV CD and UV-Vis spectroscopic studies

**DOI:** 10.1007/s00210-024-03471-3

**Published:** 2024-09-30

**Authors:** Wojciech Rogóż, Aleksandra Owczarzy, Karolina Kulig, Małgorzata Maciążek-Jurczyk

**Affiliations:** Department of Physical Pharmacy, Faculty of Pharmaceutical Sciences in Sosnowiec, Medical University of Silesia in Katowice, 40-055 Katowice, Poland

**Keywords:** Near-UV CD spectroscopy, UV-vis spectroscopy, HSA, Ligands

## Abstract

Spectroscopic methods offer many new opportunities to study protein–ligand interactions. The aim of this study was to evaluate the possibility of using near-UV CD as well as UV-Vis spectroscopic techniques to study the interaction between human serum albumin (HSA) and markers of Sudlow’s site I (warfarin, phenylbutazone) and II (ketoprofen, ibuprofen), as well as prednisolone and indapamide. In order to perform the planned measurements, near-UV CD spectropolarimetry and UV-Vis spectrophotometry have been used. It has been demonstrated that both techniques allow for rapid evaluation of non-covalent interactions between HSA and ligand, as well as identification of the HSA aromatic amino acid residues involved in this process. The near-UV CD spectroscopic data were more valuable than the analysis based on the second derivative of differential UV-Vis absorption spectra, especially for ligands with a non-specified binding site and low affinity towards HSA, such as prednisolone. The combination of both techniques makes it possible for comprehensive analysis of the interaction between HSA and ligands.

## Introduction

In the human body, the serum protein components are responsible for the transport of endogenous and exogenous substances (Tillement et al. 1979, Rabbani and Ahn 2019). There are many tools to analyze the interaction between serum proteins and ligands, for example, spectroscopic and calorimetric methods. They can provide general information about ligands-proteins interaction (Muller et al. 1994, Ross and Subramanian 1981). According to Watanabe et al., only the free (unbound) fraction of the drug in the human serum is responsible for the drug’s therapeutic effects. A free fraction of the drug can diffuse from plasma to tissue and consequently interact with pharmacological target proteins (Watanabe et al. 2018). The interaction between plasma proteins (such as human serum albumin, HSA) and ligands usually leads to changes (or modifications) in proteins’ conformation, at the level of proteins’ secondary (Tayyab et al. 2016) and tertiary (Ràfols et al. 2014) structures.

The concentration of HSA under physiological conditions varies between 35 and 50 g/l, and its molecular weight is approximately 66,500 Da (Rabbani and Ahn 2019). HSA plays a number of important functions in the human blood. Among others, it is involved in the transport of endogenous (hormones, fatty acids, metabolites, and metals) and exogenous (drugs and toxins) compounds. In addition, it regulates osmotic pressure (Yamasaki et al. 2013). HSA exerts specific antioxidant functions, and 40–80% of total HSA’s antioxidant activity can be associated with the single, free sulfhydryl group (thiol group; -SH) of cysteine amino acid residue at position 34 (Cys-34) and methionine residues (Rabbani and Ahn 2019, Khramtsov et al. 1989, Anraku et al. 1830). The HSA primary structure is formed by non-glycosylated chain of 858 amino acid residues (Assaran Darban et al. 2017, Rabbani and Ahn 2019, Yazdani et al. 2022). The secondary structure of HSA is formed by the dominant α-helix structure (Mohammadi et al. 2024) while the HSA tertiary structure consists of three homologous domains: I (residues 1–195), II (residues 196–383) and III (residues 384–585) (Rabbani and Ahn 2019, Meloun et al. 1975, Guizado 2014). Each domain is divided into two subdomains: A and B (Rabbani and Ahn 2019, Meloun et al. 1975, Guizado 2014). Subdomains IIA and IIIA are the locations of Sudlow’s site I (warfarin binding site in subdomain IIA) and Sudlow’s site II (benzodiazepine binding site in subdomain IIIA) (Sudlow et al. 1975, Sudlow et al. 1976). Sudlow’s site I is larger than Sudlow’s site II, and it can be divided into three overlapping binding regions: Ia (warfarin region; markers: warfarin, acenocoumarol), Ib (azapropazone region; markers: phenylbutazone, DNSA), and Ic (markers: n-butyl p-aminobenzoate and iodipamide) (Yamasaki et al. 2013, Yamasaki et al. 1996). The binding pocket of both binding sites is hydrophobic, which allows them to bind weakly water-soluble ligands (Rabbani and Ahn 2019). In Sudlow’s site I, strong hydrophobic interactions are usually present, while in Sudlow’s site II, the dominant types of interactions are dipole–dipole, van der Waals, and/or hydrogen bonds (Bakaeean et al. 2012). Interaction at the molecular level between protein and ligands affect their transport. On the other hand, the formation of a protein–ligand complex can have various consequences, such as reducing ligands degradation or modifying their properties (Mehrabi et al. 2022).

Circular dichroism (CD) spectroscopy allows analyzing the interaction between ligands and proteins, as well as conformational and thus functional changes in HSA structure (Kelly and Price 2000, Ranjbar and Gill 2009). Based on the CD spectra in the wavelength range between 200 and 250 nm (far-UV spectral region), it is possible to estimate the secondary structure of proteins, for example, the percentage content of α-helix and β-sheet (Ranjbar and Gill 2009). On the other hand, based on the CD spectra in the wavelength range from 250 to 315 nm (near-UV spectral region), it is possible to analyze the protein conformational and functional changes in the regions of aromatic amino acid residues, such as phenylalanine (Phe; 31 amino acid residues in HSA), tyrosine (Tyr; 18 amino acid residues in HSA), and tryptophan (Trp; 1 amino acid residue in HSA) (Ranjbar and Gill 2009, Zsila 2013).

UV-Vis spectrophotometry is also a commonly used tool that allows for the analysis of proteins and their interactions with ligands at the molecular level (Ren et al. 2019). The effect of ligand binding by HSA on its conformation can be determined based on the second derivative of the differential absorption spectra of HSA samples and their mixtures with ligand(s). Significant differences in the second derivative of the differential spectra in the wavelength range between 250 and 270 nm confirm the changes in the environment of phenylalanyl amino acid residues. In turn, differences in the second derivative of the differential spectra at wavelengths longer than 270 nm confirm the changes in the environment of tyrosyl and tryptophanyl residues (Ichikawa and Terada 1979, Terada et al. 1984).

The use of more than one spectroscopic technique and mathematical models provides complementary information about the interactions between HSA and ligand(s) (Chen et al. 2013, Assaran Darban et al. 2017, Farajzadeh-Dehkordi et al. 2022; Farajzadeh-Dehkordi et al. 2023, Fatahian Bavandpour et al. 2024). The authors aimed to confirm the hypothesis that it is possible to obtain using CD spectroscopy, in the wavelength range from 250 to 310 nm (near-UV), detailed data about the interaction between ligands and HSA. Moreover, as a comparative method, the second derivative of differential absorption spectra was used. The innovative element of this work was to demonstrate the potential application of near-UV CD spectroscopy on such a large region and to express the new perspectives on using both CD and UV-Vis spectroscopy. In the present work, four selected binding site markers, as well as two drugs, for which the HSA binding site was not precisely determined in the literature, were used. The obtained results increase the current knowledge and can be useful for other researchers.

## Materials and methods

### Chemicals

Human serum albumin (HSA) fraction V (Lot No. 4971K) and ketoprofen (KET; Lot No. 7213J) were purchased from MP Biomedicals. Methanol, ibuprofen (IBU; Lot No. 26H1368), warfarin (WAR; Lot No. 5153-025), indapamide (IND; CAS No. 26807-65-8), and phenylbutazone (PHB; Lot No. 78H1117) were obtained from Sigma Aldrich while prednisolone (PRE; Series No. 108396) was gained from Pharma Cosmetic. Dipotassium hydrogen phosphate, pure p.a. (K_2_HPO_4_) and sodium dihydrogen phosphate (NaH_2_PO_4_) were from Eurochem BGD Sp. z o. o. All chemicals were of analytical grade and used without further purification.

### Methods

Phosphate buffer (0.05 M, pH 7.4) and methanol have been used as solvents for HSA and ligands, respectively. In the spectropolarimetric measurements (near-UV CD), the ligand stock solutions concentration was 1 × 10^−2^ mol/dm^3^ and the concentration of HSA was 2 × 10^−5^ mol/dm^3^ (HSA:ligand 1:5 molar ratio). In turn, in the spectrophotometric measurements, the ligand stock solutions concentration was 5 × 10^−3^ mol/dm^3^, and the concentration of HSA was 1 × 10^−5^ mol/dm^3^ (HSA:ligand 1:5 molar ratio). The volume ratio of methanol to phosphate buffer (MeOH:phosphate buffer) was constant and less than 1:100 (v:v). All measurements were performed after 90 min of incubation of the HSA–ligand mixtures.

To investigate the HSA’s conformational changes under the influence of ligands based on the near-UV CD spectra, the JASCO J-1500 Spectropolarimeter (JASCO International CO., LTD., Hachioji, Tokyo, Japan) was used. The near-UV CD spectra were obtained in the wavelength range from 250 to 310 nm, using 10-mm path length quartz cuvettes (sample volume: 3 ml; scanning speed: 50 nm/min; temperature 298 K with an accuracy of ± 0.05 K; wavelength accuracy: ± 0.2 nm (250 to 500 nm); wavelength reproducibility: ± 0.05 nm (163 to 500 nm)).

JASCO V-730 Spectrophotometer (JASCO International Co., Ltd., Hachioji, Tokyo, Japan) was used to determine the absorption spectra of HSA and the absorption differential spectra of HSA mixtures in the presence of ligands in the range of wavelengths from 240 to 320 nm. Quartz cuvettes of path length 10 mm were used (sample volume: 3 ml; scanning speed: 40 nm/min; temperature 298 K). Based on the differential absorption spectra of the analyzed samples, the second derivative of the differential absorption spectra was determined (algorithm: Savitzky-Golay; data points: 25). The *r* parameter was determined by Eq. (1) (Ichikawa and Terada 1979, Terada et al. 1984):

1 $$r= \frac{a}{b}$$

where *a*, *b* were the values of two adjacent peaks (*a* = *a*_1_–*a*_2_; *b* = *b*_1_–*b*_2_) selected from among the peaks recorded for two wavelength ranges (250–270 nm and 270–310 nm).

### Statistics

All measurements were repeated three times. The results of spectropolarimetric measurements have been presented as near-UV CD spectra of the tested samples. In turn, the results of spectrophotometric measurements have been presented as absorption differential spectra and as mean ± relative standard deviations (mean ± SD). In order to analyze the obtained results, OriginPro software version 8.5 SR1 (Northampton, MA, USA), Microsoft Excel 2013 (Redmond, Washington, USA) and Spectra Manager Version 2.13.00 2002-2015 (JASCO International CO., LTD., Hachioji, Tokyo, Japan) were used.

## Results and discussion

**Fig. 1:**
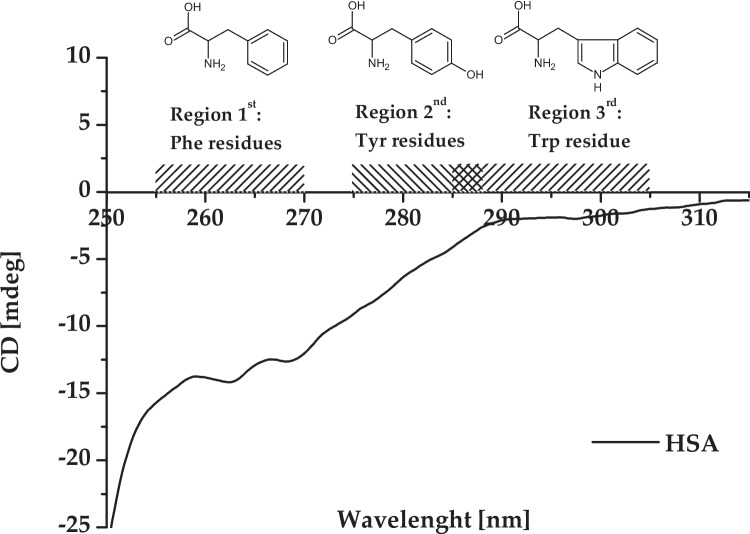
Near-UV CD spectrum of HSA (2 × 10^−5^ mol/dm^3^) in the wavelength range of 250 to 310 nm. The spectral regions that correspond to the particular amino acid residues (Phe, Tyr, Trp) are indicated

There are a lot of methods used to study the impact of ligand on protein structure, for example spectrofluorimetry (SFM), UV–Vis spectrophotometry and calorimetry (Ross and Subramanian 1981, Ràfols et al. 2014, Aki and Yamamoto 1994, Rogóż et al. 2022, Rogóż et al. 2023a, b). Nevertheless, all of them have certain limitations, which can be related to the type and the chemical nature of solvents, concentration ranges required, as well as the cost and the measurements time. Potential measurement errors can be eliminated only by using the combination of several methods or techniques. CD spectroscopy is a useful and innovative technique to study the interaction between macromolecules (proteins) and ligands (for example, drugs). Detailed information about HSA conformation can be obtained with the help of near-UV CD spectroscopy (Fig. 1) (Ranjbar and Gill 2009, Zsila 2013).

HSA’s fingerprint in the near-CD spectrum is observed in strictly defined wavelength ranges. It is induced by the aromatic amino acid residues, which are located in the protein molecule. Signals between 255 and 270 nm are caused by the phenyl group of Phe residue side chains, signals between 275 and 287 nm are connected with the Tyr amino acid residues (phenolic group in side chain), in turn, signals between 285 and 305 nm are attributable to Trp residue (indole ring in side chains) (Kelly and Price 2000, Ranjbar and Gill 2009, Zsila 2013). However, disulfide bridges signals are present throughout the near-UV CD spectrum. They can be detected in the part of the higher ellipticity value from 295 to 315 nm (Fig. 1) (Ranjbar and Gill 2009, Zsila 2013). This part of the HSA’s near-UV CD spectrum has been called by Zsila et al. the “broad negative tail” (Zsila 2013). As can be seen in Fig. 1, HSA’s near-UV CD spectrum can be divided into three distinct regions. Two distinct minima at 262.6 nm and 268.6 nm wavelengths have been identified in region 1st (due to the presence of Phe amino acid residues). Region 2nd is characterized by a “slight bulge” in the wavelength range between 280 and 283 nm, associated with Tyr residues. In region 3rd (285–305 nm), an almost straight line with a constant negative CD value is observed (Muzammil et al. 1999, Kabir et al. 2016, Musa et al. 2021).

Near-UV CD spectroscopy has been used as a helpful tool for rapid identification of the changes in the environment of individual aromatic amino acid residues (Phe, Tyr, Trp) in the HSA molecule under the influence of ligands (in HSA-ligand complexes). In this work, markers for Sudlow’s site I (phenylbutazone PHB, warfarin WAR), Sudlow’s site II (ibuprofen IBU, ketoprofen KET), as well as two other ligands (indapamide IND, prednisolone PRE) have been used (Muller et al. 1994, Ràfols et al. 2014, Yamasaki et al. 2013, Sudlow et al. 1975, Sudlow et al. 1976, Yamasaki et al. 1996, Dubois et al. 1994). The use of binding site markers enables us to indicate changes in HSA’s near-UV CD spectra. The detected differences emerged under the influence of ligand binding at different and well-known locations (Sudlow’s site I or II). In turn, IND and PRE have been chosen due to their low affinity for HSA and binding with this protein at nonspecific binding site (Boudinot and Jusko 1984, Urien et al. 1988). The interactions of these drugs (IND and PRE) with HSA are not protein-specific binding sites in the recent literature, so this study is novel. This allowed us to confirm the existence of changes in the HSA’s near-UV CD spectra under the influence of ligands, even in cases of weak binding.

**Fig. 2:**
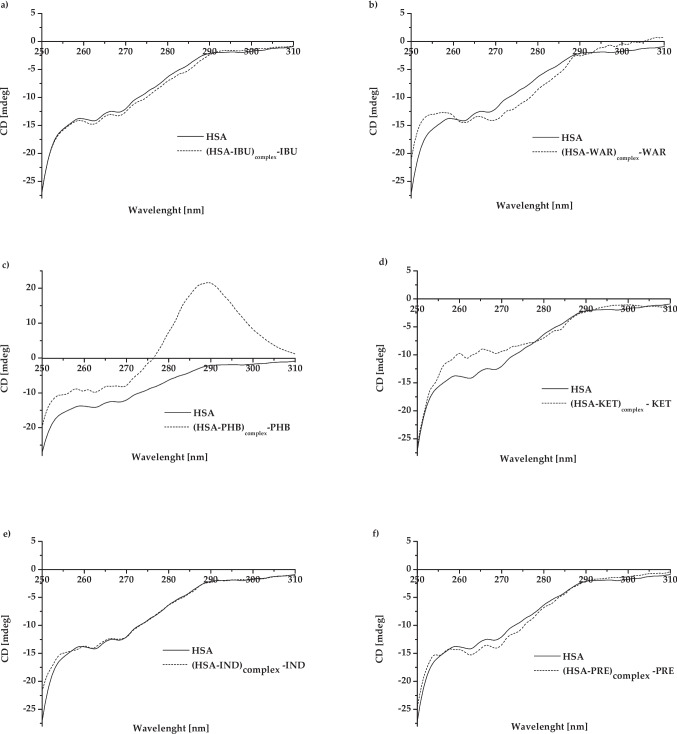
The near-UV CD spectra of HSA, both in the absence (“HSA”) and under the influence of ligands, defined in the picture as **a** “(HSA-PHB)_complex_ - PHB”, **b** “(HSA-WAR)_complex_–WAR”, **c** “(HSA-IBU)_complex_ – IBU”, **d** “(HSA-KET)_complex_ – KET”, **e** “(HSA-IND)_complex_ – IND”, and **f** “(HSA-PRE)_complex_ – PRE” at HSA:ligand 1:5 molar ratio. The near-UV CD spectra of defined as “(HSA-ligand)_complex_ – ligand” were obtained as a result of ligand`s spectrum subtraction from the mixture of (HSA-ligand)_complex_ spectrum

The diagrams below (Fig. 2a–f) illustrate the near-UV CD spectra obtained for HSA, both in the absence (“HSA”) and under the influence of ligands (“(HSA-ligand)complex–ligand”).

Based on Fig. 2, slight differences in the specific regions (from 1st to 3rd) of near-UV CD spectra have been observed between samples in the absence (“HSA”) and presence of ligand (“(HSA-ligand)_complex_ – ligand”) as the result of interaction between ligand and protein. As shown by Dockal et al., a decrease or increase in the near-UV CD spectral signals is usually connected with conformational and functional changes at the level of HSA’s tertiary structure. An increase in ellipticity at the wavelengths range between 250 and 280 nm, as well as slight decrease in ellipticity at wavelengths range between 290 and 300 nm, is consistent with decrease in protein tertiary structure and reduce its conformational packing. Dockal et al. have observed the effect of high (9.0) and low (2.0, 4.0) pH on HSA conformation (at the same concentration as in this work) based on the changes in the value of HSA ellipticity in the wavelength range between 250 and 340 nm (CD spectral signals) (Dockal et al. 2000). Kabir et al. have also demonstrated the applicability of CD spectroscopy in the studies of drug–HSA binding (Kabir et al. 2016). The aim of their study was to characterize the binding of lapatinib (an anticancer drug) to HSA, using spectroscopic methods. Based on the far- and near-UV CD spectroscopy, they showed, that lapatinib has an impact on the secondary and tertiary structures of HSA. Moreover, based on the three-dimensional fluorescence spectra, they confirmed the existence of some perturbation in the regions close to the Trp and Tyr residues (Kabir et al. 2016). In turn, Musa et al. analyzed the interaction of an antimalarial drug (lumefantrine, LUM) with HSA. They have observed, that if the LUM:HSA molar ratio increases, the CD spectral signals decrease (“decrease in the CD signal” means the same as “reduction in the spectral signals” (Kabir et al. 2016, Musa et al. 2021) or “increase in the ellipticity” (Dockal et al. 2000, Barnes et al. 1972)). Simultaneously, they have noted that LUM (in the LUM-HSA_complex_) generates some perturbation in HSA’s tertiary structure. They have also concluded that Sudlow’s site I is the most important binding site for LUM (using far-UV CD, fluorescence quenching titration, and three-dimensional fluorescence techniques) (Musa et al. 2021). The changes in the shape and/or position of the near-UV CD spectrum confirm the modification of HSA structure (HSA’s conformation and thus potential functions) (Tayyab et al. 2016, Kabir et al. 2016, Musa et al. 2021, Dockal et al. 2000). A decrease in CD values implies an increase in tertiary contacts. This may be caused by an increase in the content of highly ordered secondary structures such as α-helix (Gull et al. 2007). On the other hand, an increase in ellipticity results in a decrease in protein tertiary structure (loss of tertiary structure) and a local decrease in its conformational packing (Dockal et al. 2000). These structural changes can have an impact on protein function, i.e., changes in the HSA antioxidant activity or free thiol reactivity as a consequence of ligand binding (Turell et al. 2013, Uzelac et al. 2024, Rogóż et al. 2022). The simultaneous use of multiple spectroscopic techniques (including CD spectroscopy) provides comprehensive information about albumin’s structure modification as a result of (HSA-ligand)_complex_ formation. This thesis can be confirmed by the study of Assaran Darban et al. (2017). They analyzed the interaction between HSA and two different angiotensin I-converting enzyme-inhibiting peptides from gluten hydroxylate (P4, P6). They have used spectroscopic (UV-Vis spectroscopy, spectrofluorescence, spectropolarimetry), calorimetric (nanoITC), and molecular modeling techniques. It was shown that both peptides induced protein folding and stabilized its structure by increasing α-helix content. Binding of P4 in the presence of P6 to the protein’s amino acid residues resulted in an increase in hydrogen-bonding content (Assaran Darban et al. 2017). Based on this, it was decided that the second derivative spectroscopy of differential spectra would be used as a reference technique for near-UV CD spectroscopy. Second derivative spectroscopy of differential spectra makes it possible to analyze the environmental changes around aromatic amino acid residues in the protein structure.

Based on the changes in peak shape and position from 250 to 270 nm, it is possible to draw a conclusion about changes in the environment of phenylalanyl amino acid residues (Ichikawa and Terada 1979). In turn, the changes in the second derivative of the differential absorption spectra above 270 nm are caused by the changes in the environment of tyrosyl and tryptophanyl residues (Terada et al. 1984, Rogóż et al. 2023a, b, Owczarzy et al. 2021). The parameter *r* is used to evaluate the existence of environmental differences. It defines the absorbance difference between the various peaks and troughs in the second derivatives of the differential spectra at two wavelength ranges: 250–270 nm and > 270 nm. The use of both ranges allows the detection of changes in the environment of different aromatic amino acids (Ichikawa and Terada 1979, Terada et al. 1984). If the values of the parameter *r* for the pure HSA sample are different from the *r* values calculated for the sample with a mixture of HSA and ligand, it means that the ligand affects the spatial structure in the surroundings of aromatic amino acid residues.

**Fig. 3:**
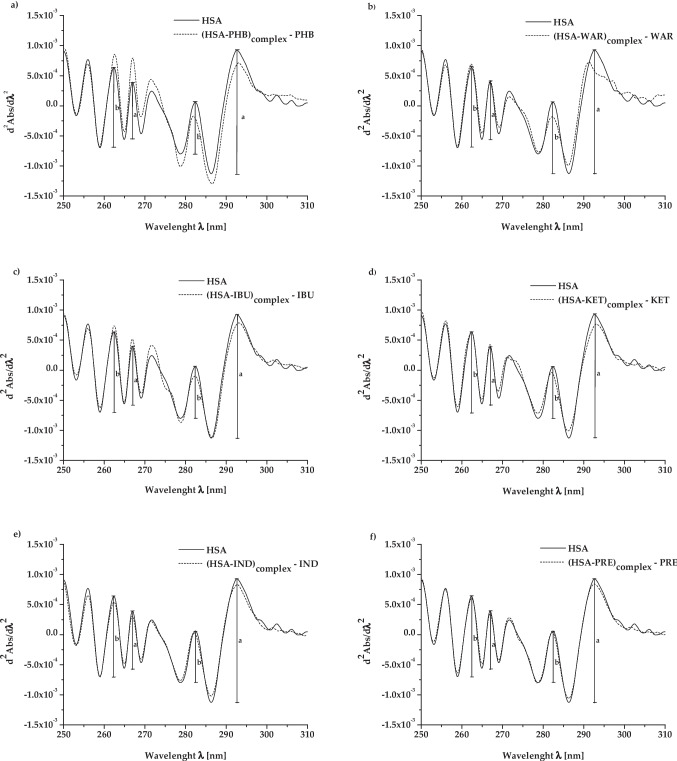
Second derivative of differential absorption spectra of human serum albumin (HSA), both in the absence (“HSA”) and presence of ligands, defined in the picture as **a** “(HSA-PHB)_complex_ - PHB”, **b** “(HSA-WAR)_complex_ – WAR”, **c** “(HSA-IBU)_complex_ – IBU”, **d** “(HSA-KET)_complex_ – KET”, **e** “(HSA-IND)_complex_ – IND”, and **f** “(HSA-PRE)_complex_ – PRE” at HSA:ligand 1:5 molar ratio; *a* and *b* are the values of two adjacent peaks (*a* = *a*_1_–*a*_2_; *b* = *b*_1_–*b*_2_)

The diagrams below (Fig. 3a–f) illustrate the second derivative spectroscopy of differential spectra obtained for HSA, both in the absence (“HSA”) and presence of ligands (“(HSA-ligand)_complex_ – ligand”). Based on the second derivative of differential absorption spectra (Fig. 3) and Eq. (1), the mean *r* values were calculated and collected in Table 1.

**Table 1:** The mean *r* values determined on the basis of the second derivative of differential absorption spectra of human serum albumin (HSA), as well as ligand–protein complexes: “(HSA-PHB)_complex_ - PHB”, “(HSA-WAR)_complex_ – WAR”, “(HSA-IBU)_complex_ – IBU”, “(HSA-KET)_complex_ – KET”, “(HSA-IND)_complex_ – IND” and “(HSA-PRE)_complex_ – PRE” at HSA:ligand 1:5 molar ratio; *λ*—wavelength

	*r* =$\frac{a}{b}$	
	*λ* 250–270 nm	*λ* > 270 nm
HSA	0.715 ± 0.026	1.805 ± 0.099
(HSA-PHB)_complex_ – PHB	0.788 ± 0.154	2.102 ± 0.183
(HSA-WAR)_complex_ – WAR	0.642 ± 0.016	2.043 ± 0.154
(HSA-IBU)_complex_ – IBU	0.618 ± 0.021	1.952 ± 0.071
(HSA-KET)_complex_ – KET	0.788 ± 0.028	2.318 ± 0.243
(HSA-IND)_complex_ – IND	0.640 ± 0.023	1.878 ± 0.072
(HSA-PRE)_complex_ – PRE	0.672 ± 0.028	1.836 ± 0.121

Based on Fig. 3, differences in the specific regions of the second derivative of differential absorption spectra between samples in the absence of ligands (“HSA”) and under the influence of ligand (“(HSA-ligand)_complex_ – ligand”) have been sighted. The smallest differences (or absence of them) were noted between the obtained spectra for samples containing IND and PRE as ligands. All included ligands (except PHB) showed the ability to interact with HSA in the regions containing phenylalanyl amino acid residues. This was evidenced by the differences in mean *r* values determined for the wavelength range from 250 to 270 nm (Table 1). At the same time, interaction between protein and ligands in HSA’s regions rich in tryptophanyl and tyrosyl amino acid residues (wavelength range above 270 nm) has been confirmed only for samples with PHB and KET (Table 1). Similar conclusions have been drawn by other authors (Rogóż et al. 2023a, b). Among others, the aim of the study was to analyze the interaction of kanamycin (KAN) and neomycin (NEO) with HSA. Calorimetric and spectroscopic techniques were used, which included the analysis of second derivative spectroscopy of differential spectra. It has been shown that KAN interacts with HSA in the region of all aromatic amino acid residues, and in turn, NEO interacts with HSA only in the region of tryptophanyl and tyrosyl amino acid residues (Rogóż et al. 2023a, b). Dieaconu et al. have also adopted this technique to study ligand-protein interaction (Dieaconu et al. 2012). The aim of their study was to investigate the binding of nickel ions (Ni^2+^) to bovine serum albumin (BSA). As can be seen from the comparison of the second derivative spectra (free BSA and BSA:Ni^2+^ 1:1 molar ratio), there are no significant changes in peak shape or position between 250 and 350 nm. However, spectral changes were observed at the wavelengths lower than 250 nm. On this basis, it was concluded that it is probably the amide groups that are responsible for the binding of nickel ions by BSA (not the aromatic amino acid residues) (Dieaconu et al. 2012). The second derivative spectroscopy of differential spectra analysis technique can be used not only to study the interaction of albumin with ligands, but also to analyze the effects of physiological (aging) (Chudzik et al. 2016) or pathological (oxidation) (Maciążek-Jurczyk et al. 2021) processes on protein structure.

Aromatic amino acids (Phe, Tyr, Trp) play a significant role in determining the properties of HSA, and the change in the spatial structure around them may indicate that the ligand studied probably also affects the properties of the protein. For example, they participate in the binding of ligands by HSA and also contribute to the catalytic activity of HSA (De Simone et al. 2021, Rabbani and Ahn 2019). According to Belinskaia et al., “The mechanism of Kemp elimination in protein molecules is associated with the presence of aromatic amino acid residues (Trp, Tyr, Phe) that provide stacking interaction with hydrogen bond donors (Lys, Arg, Ser, Tyr, His, and water molecule)” (Belinskaia et al. 2021). Importantly, both Tyr and Trp residues are present in the most important binding sites: Sudlow’s site I (subdomain IIA): Tyr-150, Trp-214; Sudlow’s site II (subdomain IIIA): Tyr-411 (Rabbani and Ahn 2019). Changes in their environment (induced by ligand binding and confirmed by differences in the *r* parameter in the range > 270 nm) probably mean that the ligand interacts with any of these sites (or several at the same time) (Rogóż et al. 2023a, b). Other Tyr residues may also influence ligand binding by HSA due to their presence in fatty acid binding sites (Tyr-138, Tyr-161, and Tyr-401) or the Cys-34 binding site (Tyr-84) (Fanali et al. 2012). The only tryptophanyl residue (Trp-214) in the primary structure of HSA is located in Sudlow site I and plays an important role in ligand binding by HSA (Fanali et al. 2012, Rabbani and Ahn 2019). Confirmation of structural changes in its environment as a result of the interaction with the ligand (different “r” values in the range > 270 nm) could mean, for example that (i) there are possible interactions of the ligand with Sudlow’s I site (Ichikawa and Terada 1979) or (ii) ligand (e.g., warfarin or ibuprofen) can affect the binding of other ligands (e.g., ascorbic acid) by HSA (Li et al. 2013).

Important conclusions also result from the confirmation of the ligand effect on structural changes in the surroundings of Phe residues (different values of “r” in the range > 270 nm). In the primary structure of HSA, Phe residues are numerous (especially compared to other aromatic amino acids: 31 Phe residues account for about 5.3% of all amino acid residues), but they are also distributed in different regions of the molecule. They are present in all HSA subunits, including both I and II Sudlow sites (Fanali et al. 2012, Rabbani and Ahn 2019). The effect of ligand binding on the Phe environment can mean (i) the existence of a stable binding between HSA and the ligand (if the ligand’s effect on Tyr and Trp residues environment is also confirmed, e.g., ketoprofen, phenylbutazone), which in turn results in limited bioavailability of the ligand (slow diffusion of the ligand from the bound fraction to the free fraction); (ii) the probable existence of only surface, unstable interactions between the ligand and the HSA (when the influence of the tested ligands on the environment of Tyr and Trp residues has not be proven, e.g., IND, PRE; more detailed information below).

In order to correctly evaluate the results obtained with the help of near-UV CD, it is necessary to compare them with the ones obtained using second derivative spectroscopy of differential spectra analysis. Therefore, in order to definitively characterize the interaction between ligands (PHB, WAR, IBU, KET, IND, PRE) and HSA, it is necessary to analyze them by simultaneous application of both techniques.

### Interaction between Sudlow’s site I markers (PHB, WAR) and HSA

Sudlow’s site I is called the phenylbutazone (PHB) and warfarin (WAR) binding site (Rabbani and Ahn 2019, Yamasaki et al. 1996, Lemli et al. 2022). PHB binds to HSA (in subdomain IIA) with a high association constant (Ka: 1.5 × 10^6^ dm^3^/mol for (HSA-KET)_complex_) (Yamasaki et al. 2013, Yamasaki et al. 1996). According to Russeva et al., electrostatic interactions are the dominant bonds between PHB and HSA, but hydrophobic bonds are also present (Russeva and Mihailova 1999). Although it is the primary binding site, there is also a secondary binding site for PHB: Sudlow’s site II in subdomain III (Rabbani and Ahn 2019). Therefore, the effect of interaction may be a reduction in the spectral signals in all analyzed regions (region 1st, 2nd, and 3rd) (Fig. 2a). The CD spectrum of HSA under the influence of PHB (“HSA” vs. “(HSA-PHB)_complex_ – PHB”) in the wavelength range from 275 to 305 nm shows a high increase. This probably indicates a loss of tertiary structure and a local reduction of its conformational packing (Dockal et al. 2000). The “slight bulge” that characterized native protein changed into an individual signal with the maximum positive value of ellipticity at 289.4 nm (Fig. 2a). It can probably be considered due to strong PHB binding to Sudlow’s site I (the Trp residue region). Chignell et al. have explained this phenomenon by describing a disturbance associated with the interaction between aromatic parts of the PHB molecule (two phenyl groups) and hydrophobic areas in the binding sites on the surface of HSA (the positive Cotton effect) (Chignell 1996). Similar results were obtained by Bertozo et al. as a result of using the induced CD (ICD) (Bertozo et al. 2018). The aim of their study was to determine and analyze the changes in PHB’s affinity towards Sudlow’s site I in HSA under the influence of oxidative alteration of Trp-214 and Lys-199 amino acid residues. They have analyzed the tertiary structure of native HSA (HSA) and oxidized HSA (HSA_OX_) as a result of the use of taurine monochloramine Tau-NHCl or taurine dibromamine Tau-NBr_2_, as well as HSA and HSAOX after binding with PHB. The maximum positive value of ellipticity at ~ 290 nm (between 280 and 300 nm) as a result of HSA binding with PHB has been observed (Bertozo et al. 2018). The shape of a PHB CD spectrum (at 289.4 nm) (induced ellipticity in PHB) is an additional evidence of the association between the analyzed ligand and HSA (Graciani and Ximenes 2013). Based on spectrophotometric analysis (Fig. 3a, Table 1), it was confirmed that PHB interacts with HSA only in the region of tryptophanyl and tyrosyl amino acid residues (in the wavelength above 270 nm). As can be deduced from the above analysis, the near-UV CD spectropolarimetry allowed for more information about the interaction between HSA and PHB than the second derivative spectroscopy of differential spectra analysis.

Warfarin, as a Sudlow’s site I marker, binds with HSA with one class of binding site (Ka: 1.40 (± 0.31) × 10^5^ dm^3^/mol for (HSA-WAR)_complex_) (Rabbani and Ahn 2019, Zsila 2013, Aki and Yamamoto 1994). Although the WAR binding to Sudlow`s site I is dominated by hydrophobic contacts, potential electrostatic (with the participation of Arg-222 and His-242) and Van der Waals interactions (with the participation of, i.e., Arg-222, Phe-223, Leu-219, and Val-241) are also possible (Petitpas et al. 2001). It was demonstrated that this ligand induces very specific changes in the near-UV CD spectral signals (Fig. 2b). Changes can be noted in the 1st region of the CD spectrum (between 255 and 270 nm; associated with Phe residues), as well as a decrease and slight increase in the ellipticity in region 2nd (wavelength range between 275 and 287 nm) and 3rd (between 285 and 305 nm; associated with Tyr and Trp residues), respectively (Fig. 2b). Especially, a decrease in CD values in region 2nd can be connected with an increase in HSA’s tertiary contacts (Gull et al. 2007). Although WAR-albumin interaction in areas rich in Phe amino acid residues is possible (Petitpas et al. 2001), their role in the binding of the ligand–protein complex is minor. This has been evidenced by the very slight changes in the region 1st near-UV CD spectrum. Also, analysis of the second derivative of differential absorption spectra allowed the conclusion that WAR interacts with phenylalanyl amino acid residues (Fig. 3b, Table 1). More important were changes in regions of near-UV CD spectrum associated with Tyr and Trp residues: the majority of tyrosines in both Sudlow’s sites I and II (mainly Tyr-150 in Sudlow’s site I) and Trp-214, located only in Sudlow’s site I (Rabbani and Ahn 2019, Yamasaki et al. 2013, Meloun et al. 1975). Based on the fluorescence analysis, it was confirmed by Sakhaei et al. that binding reaction of WAR with HSA results in a red shift of the emission maximum and a reduction of Tyr and Trp fluorescence (Sakhaei et al. 2015). This indicates some probably changes in the hydrophobicity of the fluorophore environment (Maciążek-Jurczyk 2014). Therefore, it can also be concluded that, due to the increase in the near-UV CD spectra in region 2nd, interaction of HSA with WAR can lead to significant changes in the HSA packing (Fig. 2b). On the other hand, spectrophotometric analysis (Fig. 3a, Table 1) did not unquestionably confirm that WAR interacts with the region of tryptophanyl and tyrosyl amino acid residues. The changes in peak shapes at the wavelengths above 270 nm were visible (Fig. 3b), but the values of the *r* parameters were not significantly different from each other (Table 1). An increase in ellipticity from 295 to 315 nm (the loss of the “broad negative tail”) was also observed (Fig. 2b). This may suggest an effect of WAR binding by HSA on disulfide bridges as a local reduction in the HSA’s conformational packing (Dockal et al. 2000, Ranjbar and Gill 2009, Zsila 2013). It is possible that the binding of some ligands by HSA not only leads to a change in the protein’s tertiary structure, but also influences the binding of other ligands. This is possible by changing the hydrophobicity in the environment of the binding sites, and it leads to an increase or decrease in the affinity of the HSA towards another ligand (positive or negative cooperativity, respectively) (Whitty 2008, Hunter and Anderson 2009). For example, warfarin binding by HSA (in I Sudlow’s site) significantly reduces the HSA’s ability to bind heme (in the heme-binding cleft) because both two sites are functionally linked (Ascenzi et al. 2006). On the other hand, there is no spectroscopic and functional link between II and I Sudlow’s sites, similarly as between Sudlow’s II and heme-binding cleft (Bocedi et al. 2005).

### Interaction between Sudlow’s site II markers (IBU, KET) and HSA

Ibuprofen (IBU) as well as ketoprofen (KET) are Sudlow’s site II markers (Ka: 9.0 (± 1.0) × 10^5^ dm^3^/mol for (HSA-IBU)_complex_ (Ràfols et al. 2014) and 2.79 (± 3.6) × 10^5^ dm^3^/mol/2.81 (± 4.2) × 105 dm^3^/mol for (HSA-KET)_complex_ (KET: R and S isomers, respectively) (Dubois et al. 1994)). They bind to HSA in subdomain IIIA as a primary binding site with high affinity. Simultaneously, IBU and KET can interact with HSA in other subdomains (IB, IIA) because they have more than one class of binding site (Rabbani and Ahn 2019, Muller et al. 1994, Ràfols et al. 2014, Manoharan et al. 2015, Alexandri et al. 2023).

Slight differences between the near-UV CD spectrum of “HSA” and “(HSA-IBU)_complex_ –HSA” have been observed. There were significant differences, especially in the regions where the signals come from Tyr residues (region 2nd) and, to a lesser extent, from Trp-214 residue (region 3rd) (Fig. 2c). As Zsila et al. and Rabbani et al. reported, IBU strongly interacts with albumin mainly in subdomain IIIA (Sudlow’s site II) (Ràfols et al. 2014), as well as in subdomain IB, where Tyr residues are located (Rabbani and Ahn 2019, Zsila 2013). These conclusions were confirmed by the most significant changes in the near-UV CD spectra of albumin in complex with IBU in region 2nd (Fig. 2c). The influence of a ligand may be limited not only to its effect on the structure of the main binding site, but also on the closest environment. This, in turn, can generate additional signals or interfere with those already existing. Therefore, near-UV CD spectrum of the “HSA-IBU)_complex_ – IBU” had some minor changes, similar to those observed by Zsila, as a result of using of both near-UV CD and other spectroscopic techniques (Zsila 2013). The spectrophotometric measurements proved that IBU binds to HSA in the region of phenylalanyl amino acid residues. However, it was not possible to confirm that a bond was formed between the IBU and the region of Trp and Tyr amino acid residues (Fig. 3c, Table 1). The simultaneous use of both techniques allowed us to draw final conclusions about the binding of IBU to HSA. The interaction between HSA and IBU reveals environmental changes around aromatic amino acid residues. Ketoprofen (KET) interacts with HSA mainly in subdomain IIIA, hence it is called Sudlow’s site II marker (Rabbani and Ahn 2019, Muller et al. 1994, Manoharan et al. 2015, Czub et al. 2022). Besides tyrosyl residues, phenylalanine residues are also located in Sudlow’s site II (subdomain IIIA) (Meloun et al. 1975). Therefore, the most significant differences in region 1st of near-UV CD spectra between HSA and (HSA-KET)_complex_ – KET were observed (Fig. 2d). This may indicate a local reduction of the protein tertiary structure (Dockal et al. 2000). Phe residues also occur in HSA subdomain IIA and IB (Meloun et al. 1975), but they do not participate directly in the interaction with the ligand. Binding sites in HSA contain some amino acid residues involved in the formation of a bond between protein and ligand. These are included in Sudlow’s site I: at the bottom of the pocket Tyr-150, His-242, and Arg-257; at the entrance of the pocket Lys-195, Lys-199, Trp-214, Arg-218, Arg-222, and Glu-292. In turn, these are included in Sudlow’s site II: Leu-387, Arg-410, Tyr-411, Leu-453, and Ser-489 (Rabbani and Ahn 2019, Yamasaki et al. 2013). This leads to the conclusion that HSA shows the ability to bind KET not only in subdomain IIIA (Sudlow’s site II), but also in other areas of the HSA molecule, where Phe amino acid residues are located (subdomain IIA, IB) (Muller et al. 1994, Meloun et al. 1975). The same conclusions can be drawn on the basis of the analysis of the second derivative spectroscopy of differential spectra (Fig. 3d) and the mean *r* value (Table 1). KET interacts both with the regions of phenylalanyl and tryptophanyl and tyrosyl amino acid residues. Literature data showed that KET has more than one binding site: primary (the high affinity) and secondary (the low affinity) binding sites on the surface of HSA (Guo et al. 2011, Maciążek-Jurczyk 2014, Rogóż et al. 2023a, b). Changes in HSA ellipticity under the influence of KET were also observed in the areas of the near-UV CD spectrum associated with Tyr and Trp residues (Fig. 2d). For example, Tyr-411 is very important in the formation of bonds between KET and HSA. Interaction between KET and Tyr-411 (located in HSA Sudlow’s site II) supported by both hydrophobic and hydrogen bonds has been confirmed by SFM measurements (Czub et al. 2022, Maciążek-Jurczyk 2014). HSA has more than one binding site for KET, and it has been proven by both analysis of the second derivative of differential absorption spectra and near-UV CD spectra. However, it is not possible to definitively confirm with these techniques that one of these sites is the Sudlow II site.

### Interaction between ligands with non-specified binding site and low affinity to HSA (IND, PRE) and HSA

Indapamide (IND) and prednisolone (PRE) can interact with albumin; however, the association constant of this reaction is very low (Ka: 2.5 (± 0.60) × 10^3^ dm^3^/mol (Urien et al. 1988) or 3.32 × 10^3^ dm^3^/mol (Malik et al. 2020) for (HSA-IND)_complex_; 3.7 (± 0.33) × 10^3^ dm^3^/mol/1.1 ± 0.09 × 10^3^ dm^3^/mol (depending on temperature) (Pontremoli et al. 2018) or approximately 1.0 × 10^3^ dm^3^/mol (Boudinot and Jusko 1984) for (HSA-PRE)_complex_). The van der Waals forces, or hydrogen bonds, are mainly responsible for stabilizing the complex of BSA with PRE (Shi et al. 2013). On this basis, it can be assumed that they are also significant for the interaction between PRE and HSA. Very slight but significant changes in ellipticity in the range of near-UV CD associated with Phe (between 255 and 270 nm) under the influence of IND have been observed. In turn, the presence of PRE induces changes in the range of near-UV CD associated with Phe and Tyr (wavelengths range from 255 to 270 nm and from 275 to 287 nm, respectively; Fig. 2e) amino acid residues (region 1st and 2nd). This phenomenon has been explained by Bertozo et al. as the change in the degree of protein packing in regions surrounding the Phe and Tyr amino acid residues (Bertozo et al. 2018). The influence of “mild oxidizing reagents” like taurine dibromamine (Tau-NBr_2_) located in Sudlow’s site I Trp-214 and Lys-199 amino acid residues was analyzed. It has been demonstrated that the presence of Tau-NBr_2_ can lead to a small blue shift and therefore change the packing of the HSA molecule (based on the spectrofluorimetric measurements). Simultaneously, they have observed an increase in HSA near-UV CD spectral signals under the influence of Tau-NBr_2_ (Bertozo et al. 2018). Analysis of the second derivative of differential absorption spectra leads to the conclusion that both IND and PRE interact with the regions of phenylalanyl amino acid residues. However, it is not possible to confirm that any of the tested drugs can interact with the regions of tryptophanyl and tyrosyl amino acid residues (Fig. 3e, f; Table 1).

The greater differences in the near-UV CD spectra of a protein do not always correspond with a higher affinity of the ligand for the binding macromolecule. This can be confirmed by observations based on Fig. 2e and f. Although the affinity of IND towards HSA is higher than that of PRE, greater differences towards the spectrum of pure HSA were observed for PRE than IND (Fig. 2e, f) (Boudinot and Jusko 1984, Urien et al. 1988). Each of the applied techniques allowed us to confirm that some interaction between HSA and both included ligands was possible. The results of using these two techniques were consistent with the interaction of the tested drugs with the regions of the Phe amino acid residues. However, only near-UV CD was allowed to confirm the existence of PRE interactions with regions with Tyr amino acid residues. On this basis, it can be concluded that near-UV CD spectropolarimetry was a more precise technique than the analysis of the second derivative of differential absorption spectra. The validity of multispectroscopic analysis of ligand–HSA interaction was also confirmed by Farajzadeh-Dehkordi et al. (2023). The aim of their work was the biophysical analysis of HSA interactions with the potentially toxic azo dye Reactive Yellow 145 (RY145). For this purpose, they used spectroscopic measurements and in silico analysis. As they showed, the protein–ligand complex was formed spontaneously and stabilized by hydrogen bonds and van der Waals interactions (SFM). The authors also confirmed that RY145 induced an increase in hydrophobicity in the region near the Tyr and Trp amino acid residues (UV-Vis spectroscopy), as well as a significant decrease in the α-helix structure content of HSA (far-UV CD spectroscopy) (Farajzadeh-Dehkordi et al. 2023).

## Conclusions

Near-UV CD spectroscopy is a very useful and effective research technique. It was found that near-UV CD separately allows for preliminary, rapid evaluation of ligand binding to HSA. The analysis of near-UV CD spectra divided into individual regions makes it possible to rapidly identify both the interaction and the predictable regions (environment around Phe, Tyr, and Trp amino acid residues) of interaction between ligand(s) and HSA at the level of preliminary studies. However, to draw more specific conclusions, other methods of measurement can be helpful, like, for example, the analysis of the second derivative of differential absorption spectra. The combination of both techniques allows for a comprehensive analysis of the interaction between HSA and ligands. Near-UV CD does not always allow evaluating the binding site(s) on HSA. What’s more, the use of near-UV CD makes it possible to analyze the ligands connected with both high (like Sudlow’s sites I and II markers) and low (like IND or PRE) affinity binding sites.

## Data Availability

The datasets used and/or analyzed during the current study are available from the corresponding author on reasonable request.
